# Gap difference in navigated TKA: a measure of the imbalanced flexion-extension gap

**DOI:** 10.1051/sicotj/2018007

**Published:** 2018-07-13

**Authors:** Zi-Yang Chia, Hee-Nee Pang, Mann-Hong Tan, Seng-Jin Yeo

**Affiliations:** Department of Orthopaedic Surgery, Singapore General Hospital, 20 College Road, Academia, Level 4, Singapore 169865 Singapore

**Keywords:** Flexion extension gaps, Gap balancing, Navigated total knee arthroplasty, Computer assisted knee arthroplasty

## Abstract

*Introduction*: The success of Total Knee Arthroplasty (TKA) hinges on balanced flexion-extension gaps. This paper aims to evaluate the correlation between imbalanced gaps and clinical outcomes, and hence help quantify the imbalanced gap in navigation-assisted total knee arthroplasty.

*Methods*: We studied 195 knees with an average follow-up of two years. Flexion-extension gaps were obtained from computer calculation upon cementation of implants in both flexion (90°) and extension. The gap difference (GD) was defined as the measured difference between the gaps in flexion and extension.

*Results*: At 2 years after surgery, the mean ROM in the balanced group, with GD less than or equal to 2 mm, was 115.1° ± 16.6° and the mean ROM in the imbalanced group was 116.7° ± 12.1°. This was not statistically significant with *p*-value 0.589. Balanced flexion-extension gaps also did not show significant difference in terms of mechanical alignment, with 0.29 ± 0.89 in the balanced group at 2 years, and 0.65 ± 1.51 in the imbalanced group with *p*-value 0.123. Balanced gaps however, were associated with improved outcomes in terms of physical functioning, bodily pain, social functioning, Oxford and Knee scores at 6 months and improved social functioning scores at 2 years.

*Conclusions*: Computer navigation is a useful tool for assessing the gap balance in TKA. Balanced flexion-extension gaps, with gap differences of less than or equal to 2 mm, is associated with improved clinical outcomes at 6 months.

## Introduction

Restoration of the mechanical axis and balanced flexion-extension gaps are factors for a successful total knee arthroplasty (TKA) [1,2]. Gap balancing influences the final knee kinematics [3] and incorrect soft tissue balancing can contribute to accelerated polyethylene wear [4]. Thus, balanced extension and flexion gaps are important [5]. Extension balance is affected by ligamental releases and flexion balance is affected by suitable collateral and posterior cruciate ligament (PCL) tension [6]. However, the optimal soft tissue balancing in conventional TKA is a perennial challenge as it is often an intuitive process [2], in particular the amount of PCL recession in cruciate-retaining (CR) TKA [7]. Computer navigation has been shown to be useful for correction of alignment [8] and more precise placement of implants in TKA [9]. It can also provide quantitative data to assess the flexion and extension gaps [10].

The current literature described the effects of non-rectangular gaps on clinical outcomes [11–15]. As yet, there are few reports on the effects of flexion-extension gap asymmetry on clinical outcomes. We hypothesized that asymmetry between flexion and extension gaps, adversely affects outcomes when it goes beyond a certain threshold. The aim of this study was to evaluate the correlation between flexion-extension gap asymmetry and clinical outcomes in computer assisted total knee arthroplasty.

## Materials and methods

### Study design

Computer navigated TKA was performed on 195 consecutive patients (195 knees) in this prospective study after approval was sought for our study protocol from our hospital's ethics committee. The consent of all participating patients was obtained. The inclusion criterion was primary osteoarthritis of the knee. The exclusion criteria included patients with previous knee surgery, rheumatoid arthritis, infection and those who were unsuitable for unconstrained TKA. There were 155 females and forty males, with a mean age of 66.6 years (range, 50 to 90 years). The surgeries were done by the two senior surgeons (YSJ and TMH).

### Operative procedure

Eighty three cruciate-retaining and 112 posterior stabilized (PS) TKAs were performed using metal-backed fixed-bearing tibial prostheses (PFC, Depuy Orthopaedic International, Leeds, UK). All patellae were resurfaced.

General or spinal anaesthesia was employed. 147 patients underwent spinal anaesthesia and 48 patients underwent general anaesthesia. Each surgery was done with the aid of a tourniquet after intravenous prophylactic antibiotic. The standard medial parapatellar approach was used with patellar eversion. All femurs were prepared after the tibiae, and the duration of the surgery was recorded at the end of the surgery.

The software used for the navigation assisted surgery was Ci Mi TKR Version 2.0 by BrainLab/ Depuy Orthopaedic Inc (Johnson and Johnson, Leeds, UK). Anatomical landmarks were registered with dual 3 mm unicortical pins inserted at a distance from the surgical approach, and a pointer with infrared reflectors. The tibial cut was performed first. This was followed by the femur cuts and ligament balancing. Soft tissue releases were done to produce a rectangular gap at 0 degrees extension and the space between the distal femur and proximal tibia was recorded. Subsequently, the knee was flexed to 90 degrees and the space between the posterior femoral condyles and proximal tibia stored. The use of navigation is beneficial as it helps to quantify the soft tissue tension in the gap balancing technique. The size and the position of the femoral component was adjusted on a virtual computer model to achieve equal extension and flexion gaps. Final flexion and extension gaps were then recorded after implantation of tibial and femoral components. The Gap Difference (GD) was determined by obtaining the difference between the flexion gap and the extension gap.

Our study demonstrated that increasing Gap Differences correlated with poor clinical outcomes. To find a specific Gap Difference threshold, the patients were divided into 2 groups based on varying Gap Differences. Sequential tests were then conducted with different GDs until the minimum value with statistical significance was obtained, which was found to be 2 mm. Final flexion-extension gap difference was thus defined as a difference in flexion and extension gaps of more than 2 mm. Group A patients had Gap Differences of equal or less than 2 mm, and Group B patients had Gap Differences of more than 2 mm.

Each patient had graduated compression stockings and intermittent pneumatic calf pumps as prophylaxis against deep vein thrombosis. All patients had similar post operative analgesics regime and underwent the same rehabilitation protocol.

### Outcome measures

The preoperative and outcome measures were evaluated by staff from our Orthopaedic Diagnostic Center at six months and two years postoperatively. The range of motion of each operated knee was assessed using a goniometer. The reading was repeated three times and the average measurement was used. Alignment was similarly measured in weightbearing at 6 months and 2 years during follow up.

The preoperative and postoperative functional status of the patients were evaluated using the Knee Society Clinical Rating Score [12], Oxford Knee Questionnaire [8] and SF-36 Health Survey.

### Statistical analysis

Statistical analysis was performed with SPSS statistical software (version 17.0; SPSS, Chicago, Illinois). Univariate analysis was performed with Chi-square or the Fisher's exact test for comparison of proportions between two categorical data. The *t*-test was used to compare the parametric data between two independent samples. A *p*-value < 0.05 was considered significant.

## Results

The patients were divided into 2 groups, group A with Gap Differences less than or equal to 2 mm, and Group B with Gap Differences more than 2 mm. Group A had mean (SD) age of 66.1 ± 8.0 comprising 26 males and 98 females, whereas Group B had a mean (SD) age of 67.4 ± 7.0 years comprising 14 males and 57 females. There was no significant difference between the pre-operative alignment and the pre-operative ROM when comparing Group A to Group B. The length of stay, duration of surgery, proportion of CR vs PS knees also showed no significant difference between both groups.

### Intra-operative gap measurements

Group A had a mean extension gap of 12.0 mm ± 1.9, and a mean flexion gap of 11.6 mm ± 2.3. Group B had a mean extension gap of 12.9 mm ± 2.7, and a mean flexion gap of 10.7 mm ± 3.6. The mean Gap Difference in Group A was 1.3 ± 0.6, and 3.8 ± 1.5 in Group B (Table 1).

**Table 1:** Comparison of clinical measurements.

	Group A (GD less than or equal to 2 mm)	Group B (GD more than 2 mm)	*p*-value
Range of motion (mean ± stand. dev.) (degrees)			
Preoperative	112.3 ± 22.865	112.143 ± 20.766	0.962
Postoperative at 6 months	109.902 ± 15.728	106.906 ± 16.319	0.232
Postoperative at 24 months	115.061 ± 16.577	116.7 ± 12.107	0.589
Mechanical axis (mean ± stand. dev.) (degrees)			
Preoperative	1.92 ± 4.605	2.47 ± 4.8	0.431
Postoperative at 6 months	0.45 ± 1.535	0.55 ± 1.511	0.675
Postoperative at 24 months	0.29 ± 0.89	0.65 ± 1.511	0.123

### Range of motion

In Group A, the ROM improved preoperatively from 112.3° ± 22.9° to 115.1° ± 16.6° at 2 years (*p* = 0.149). In Group B, the ROM improved preoperatively from 112.1° ± 20.8° to 116.7° ± 12.1° at 2 years (*p* = 0.693).

### Alignment

The alignment in Group A improved from pre-operative coronal plane alignment of 1.92° ± 4.605°, to 0.29° ± 0.9° at 2 years (*p* = 0.00). The alignment in Group B improved from pre-operative coronal plane alignment of 2.5° ± 4.8°, to 0.7° ± 1.5° at 2 years (*p* = 0.007).

### Knee society scores and oxford knee scores (Table 2)

Group A patients showed improved outcomes in clinical scores, such as the Knee Society Score and Oxford Knee Score (OKS), at 6 months with significance. However, the outcomes for both groups were comparative with no significance at 2 years. At 6 months post-operation, the proportion of patients who had excellent results in the Knee Score (score ≥ 80) in Group A was 79.6%, as compared to 64.0% in Group B (*p* = 0.032). In terms of Function Scores, Group A had 42.2% with excellent results, while Group B had 33.3% (*p* = 0.27). The proportion of patients who had excellent results in OKS (score < 24) in Group A was 91.4%, as compared to 74.2% in Group B (*p* = 0.004). At 2 years post-operation, the proportion of patients who had excellent results in the Knee Score (score ≥ 80) in Group A was 86.6%, as compared to 87.5% in Group B (*p* = 0.570). In terms of Function Score, Group A had 50% of patients with excellent scores, and Group B had 40% (*p* = 0.210)The proportion of patients who had excellent results in OKS (score < 24) in Group A was 91.2%, as compared to 85.0% in Group B (*p* = 0.248).

**Table 2:** Comparison of clinical data.

	Group A (GD less than or equal to 2 mm)	Group B (GD more than 2 mm)	*p*-value
Knee score (mean ± stand. dev.)			
Preoperative	39.187 ± 21.041	38.681 ± 17.548	0.859
Postoperative at 6 months	83.876 ± 9.695	77.644 ± 16.758	0.010
Postoperative at 24 months	87.026 ± 10.690	83.741 ± 15.909	0.161

Function Score (mean ± stand. dev.)			
Preoperative	56.138 ± 17.436	54.058 ± 20.62	0.459
Postoperative at 6 months	70 ± 15.628	65.167 ± 19.614	0.084
Postoperative at 24 months	74.740 ± 19.295	67.870 ± 21.070	0.056

Oxford Knee Score (mean ± stand. dev.)			
Preoperative	33.943 ± 8.202	35.275 ± 8.881	0.296
Postoperative at 6 months	18.933 ± 4.74	22.783 ± 8.853	0.003
Postoperative at 24 months	18.376 ± 4.721	20.0926 ± 6.983	0.120

SF-36 Score (mean ± stand. dev.)			
Physical Functioning (PF)			
Preoperative	41.179 ± 21.596	22.826 ± 36.314	0.644
Postoperative at 6 months	65.809 ± 19.723	57.666 ± 22.819	0.017
Postoperative at 24 months	69.350 ± 18.413	62.314 ± 23.963	0.073

Role-Physical (RP)			
Preoperative	28.252 ± 39.717	35.159 ± 18.356	0.350
Postoperative at 6 months	69.762 ± 40.975	55.833 ± 45.185	0.045
Postoperative at 24 months	70.779 ± 38.984	62.037 ± 42.819	0.227

Bodily Pain (BP)			
Preoperative	36.26 ± 18.995	35.159 ± 18.356	0.697
Postoperative at 6 months	66.209 ± 26.394	55.05 ± 24.823	0.008
Postoperative at 24 months	66.012 ± 23.771	61.092 ± 25.817	0.263

General Health (GH)			
Preoperative	71.2114 ± 17.928	64.405 ± 19.755	0.016
Postoperative at 6 months	73.2 ± 18.104	68.4 ± 21.607	0.129
Postoperative at 24 months	71.883 ± 20.778	68.018 ± 22.389	0.312

Vitality			
Preoperative	68.821 ± 20.725	67.826 ± 19.336	0.744
Postoperative at 6 months	75.857 ± 18.507	70.083 ± 20.284	0.650
Postoperative at 24 months	74.350 ± 17.646	70.370 ± 17.720	0.207

Social functioning (SF)			
Preoperative	61.991 ± 37.752	55.435 ± 36.03	0.242
Postoperative at 6 months	88.214 ± 25.229	69.583 ± 36.921	0.000
Postoperative at 24 months	94.318 ± 18.864	82.870 ± 31.407	0.019

Role Emotional (RE)			
Preoperative	85.095 ± 33.93	78.261 ± 39.534	0.209
Postoperative at 6 months	94.92 ± 19.492	87.778 ± 31.87	0.120
Postoperative at 24 months	96.536 ± 17.592	96.296 ± 19.062	0.941

Mental health			
Preoperative	79.317 ± 17.388	77.449 ± 17.136	0.474
Postoperative at 6 months	84.266 ± 13.024	78.8 ± 16.798	0.320
Postoperative at 24 months	84.051 ± 12.740	83.037 ± 14.452	0.672

### Complications

One patient (1.4%) in each group had superficial infection which responded to IV antibiotics, one patient in group B had distal DVT. There no revisions performed during the two year follow up.

## Discussion

Balanced flexion-extension gaps are essential for good outcomes in TKA. However it is still an intuitive process, based on subjective feeling. The use of computed navigation has improved gap balancing [16], mechanical alignment [17] and implant positioning. This paper aims to correlate gap difference with clinical outcomes, and to quantify the imbalanced flexion extension gap.

Imbalanced flexion-extension gaps results in decreased postoperative range-of-motion, as well as poorer functional scores and increased rates of implant failures [18–21]. A study performed by Hiroshi et al. [11] over 4 years reported positive correlation between ROM and gap difference. By affecting the final knee kinematics, imbalanced gaps lead to suboptimal soft tissue balance, and therefore accelerated polyethylene wear, as well as increasing likelihood of flexion deformity >4° [22]. In the event of a tight flexion gap, measures can be taken intraoperatively to balance the gap. The posterior slope of the tibia can be increased up to 7 degrees if flexion gap tightness is felt to be secondary to tightness of both collateral ligaments and the posterior cruciate ligament [6]. The posterior cruciate ligament can be released when it is determined to be excessively tight while the collateral ligaments is deemed balanced. In addition, one can consider femoral recession and conversion from CR to PS TKA [23].

Other studies have different means of gap measurement, using spacers and lamina spreaders under specific joint distraction forces [24,25]. In this study, although gaps were measured and balanced after bone morphing, final gaps as analysed here were only recorded after implantation. This was done without the use of lamina spreaders. Hence, this data is limited to correlating post-implantation gaps with clinical outcomes. It should be noted that this post-implantation gap is affected by other factors such as implant size and placement, hence it is only a surrogate marker of soft tissue balance. This study can be improved by measuring pre-implantation gaps under specific distraction forces, the results of which will have greater clinical utility in correcting imbalances before final implantation. In addition, if post-implantation gaps can be shown to have good correlation with pre-implantation gaps, then the results of this study can likewise have improved clinical significance.

We find navigation beneficial as it allows us to create rectangular gaps, minimizing medial-lateral asymmetry [16]. It also helps to balance the flexion and extension gap, and to quantify the tight flexion gap after implantation.

No studies have provided readily quantifiable values for the definition of an imbalanced flexion-extension gap. In our study, patients with imbalanced gaps of more than 2 mm difference have poorer clinical outcomes at 6 months. This is useful for intra-operative gap balancing to improve clinical outcomes.

The patients in Group A had superior Knee Scores at 6 months, which is the objective component of the Knee Society Score. This difference could be influenced by the improved ROM seen in Group A at 6 months. However, at 2 years, this difference in ROM is equalized, which could help explain the similar Knee scores at 2 years for both groups.

Some of the improvement in objective outcomes translated into improved clinical scores at 6 months, as evidenced by the superior Oxford Knee Scores in Group A. However, the differences in the OKS did not show up in the SF-36 scores. This is possibly because the OKS is knee-specific, as opposed to the SF-36 which is a marker for general health, hence differences seen in the OKS were not significant enough to show up on the SF-36 scores.

We acknowledge that given the number of patients in Group B, it may be perceived that a substantial proportion of knees are not adequately balanced. However, the 2 mm benchmark is an arbitrary threshold, and there is currently a lack of a widely-accepted definition of a balanced knee. Nonetheless, this illustrates that even with computer navigation, to produce balanced flexion and extension gaps, one has to achieve appropriate soft tissue balancing, bone cuts, and implant choice.

The effects of imbalanced gaps could be further studied by focusing on the sub-group of patients with gap differences of more than 3 mm. At the same time however, the senior authors acknowledge that the gap differences revealed by the computer navigation may not accurately reflect the intra-operative gap difference.

By incorporating the use of navigation-assisted surgery, this paper aims to quantify the imbalanced flexion-extension gap by studying 2 year outcomes. Long term studies could however be done on the effects of gap difference, and how this correlates to the difference (if any) in outcomes between navigation-assisted surgery and conventional TKRs.

Limitations. Given the relative short timeline, we were not able to gather data to demonstrate improved outcomes in the long term. Long term results will be useful to study the effects on imbalanced gaps on outcomes such as polyethylene wear.

Our study has incorporated both CR and PS type TKAs done by both surgeons alike. The controversy over the superiority of either types of TKA continues as both show no significant difference in outcomes.

We acknowledge that the gap difference measured after bone cuts, with a tensiometer, is a better indicator of soft tissue balance. This will better guide the extent of soft tissue releases required to achieve balanced gaps, hence improved clinical outcomes.

In conclusion, computer navigation is a useful tool for providing quantitative data in correlating balanced gaps with clinical outcomes. This study also demonstrated that patients with balanced flexion-extension gaps, with gap differences of less than or equal to 2 mm, show improved clinical outcomes at 6 months.

## Conflict of interest

The authors declare that they have no conflicts of interest in relation to this article.
